# Mid-term Outcomes of Transcatheter Aortic Valve Replacement *vs.* Surgical Aortic Valve Replacement in Low-to-Moderate Risk Patients with Severe Aortic Stenosis: A Systematic Review and Meta-analysis

**DOI:** 10.21470/1678-9741-2024-0250

**Published:** 2025-10-31

**Authors:** Capela António Dicazeco Pascoal, Hilária Saugo Faria, Antonino de Jesus Francisco, Clara de Andrade Pontual Peres, Luiz Fernando Tavares, Barbara Bombassaro Masiero, Mohamed Doma, Valdano Manuel

**Affiliations:** 1 Department of Medicine, Universidade Agostinho Neto, Luanda, Angola; 2 Escola de Medicina, Universidade Federal de Santa Maria, Santa Maria, Rio Grande do Sul, Brazil; 3 Department of Medicine, Universidade de Pernambuco, Recife, Pernambuco, Brazil; 4 Department of Medicine, Universidade Federal de Alfenas, Alfenas, Minas Gerais, Brazil; 5 Department of Medicine, Pontifícia Universidade Católica do Rio Grande do Sul, Porto Alegre, Rio Grande do Sul, Brazil; 6 Alexandria Faculty of Medicine, Alexandria, Egypt; 7 Complexo Hospitalar de Doenças Cardio-Pulmonares Cardeal Dom Alexandre do Nascimento, Luanda, Angola

**Keywords:** Aortic Stenosis, Transcatheter Replacement, Atrial Fibrillation, Artificial Pacemaker, Stroke

## Abstract

**Introduction:**

Several clinical trials have demonstrated the non-inferiority of transcatheter aortic valve replacement compared with surgical aortic valve replacement in patients with severe aortic stenosis and low to intermediate surgical risk. However, mid-term results are still contentious. We performed this meta-analysis to compare the safety and efficacy of transcatheter *vs.* surgical aortic valve replacement in the mid-term in patients with aortic stenosis at low to moderate surgical risk.

**Methods:**

We searched Embase, PubMed®, and Cochrane databases for randomized clinical trials that compared transcatheter with surgical aortic valve replacement in patients with symptomatic severe aortic stenosis with a follow-up of at least four years. Outcomes of interest were all-cause mortality and disabling stroke.

**Results:**

We included six randomized clinical trials encompassing 6,444 patients with severe aortic stenosis, of whom 3,282 (50.9%) underwent transcatheter aortic valve replacement. There was no difference in all-cause mortality (risk ratio [RR] 1.08; 95% confidence interval [CI] 0.94 - 1.25; *P* = 0.30) and disabling stroke (RR 0.95; 95% CI 0.75 - 1.21; *P* = 0.67) between groups. In the subgroup analysis, five-year mortality (RR 1.28; 95% CI 1.10 - 1.49) was higher in the transcatheter group. The new pacemaker implantation (RR 2.22; 95% CI 1.42 - 3.45) rate was higher in the transcatheter group. However, the new atrial fibrillation (RR 0.40; 95% CI 0.31 - 0.52) rate was higher in the surgical group.

**Conclusion:**

Mid-term mortality and disabling stroke rates in patients with severe aortic stenosis treated with either transcatheter or surgical aortic valve replacement were similar.

## INTRODUCTION

**Table:** 

Abbreviations, Acronyms & Symbols				
AS	= Aortic stenosis		RCTs	= Randomized controlled trials
CI	= Confidence interval		RR	= Risk ratio
DM	= Diabetes mellitus		SAVR	= Surgical aortic valve replacement
NA	= Data not available		STS	= Society of Thoracic Surgeons
NYHA	= New York Heart Association		TAVR	= Transcatheter aortic valve replacement

Aortic stenosis (AS), the most prevalent heart valve disease in the elderly, is characterized by a hemodynamically significant narrowing of the aortic valve and it stands as a major contributor to global morbidity and mortality^[1-3]^. Its prevalence is increasing rapidly because of the aging population, therefore, it is estimated that there are, now, more than 291,000 candidates for aortic valve replacement in North America and Europe^[4,5]^. The benefit of transcatheter aortic valve replacement (TAVR) in patients who are inoperable is already well-established^[6-8]^. Surgical aortic valve replacement (SAVR) is one of the most common cardiac procedures and it is a definitive therapy that considerably improves symptoms and long-term survival of patients with severe AS. The procedure has been the gold standard for more than 50 years, and its operational mortality has been described as low: 0.5% to 1% in specialized institutions, with promising long-term results^[9,10]^.

The perioperative risk of mortality associated with SAVR tends to increase with age, reaching up to approximately 10% in patients aged 85 to 90 years^[11]^. Although surgery is still considered an intervention of choice in patients with a low risk of surgical complications and severe AS, TAVR is continually gaining ground in the lower-risk groups^[6]^. Approximately 90% of patients undergoing aortic valve replacement are considered to be at low and moderate surgical risk^[12,13]^. Several factors are influential in this current scenario, including the high prevalence of patients requiring valve replacement and technological advances in valve replacement that allows a minimally invasive approach under local anesthesia^[14-16]^.

Although previous meta-analyses have demonstrated that TAVR is not inferior to SAVR in patients with low to moderate surgical risk, they primarily included studies with shorter follow-up periods, limiting the assessment of mid-term outcomes^[17,18]^. To address this gap, the present systematic review and meta-analysis aimed to comprehensively compare the mid-term safety and efficacy of TAVR *vs.* SAVR in patients with AS at low to moderate surgical risk by evaluating two critical endpoints: all-cause mortality and disabling stroke, using more recent evidence with extended follow-up data.

## METHODS

This systematic review with meta-analysis was registered in the *International Prospective Register of Systematic Reviews* (or PROSPERO) under protocol CRD42024501903. It was designed and conducted according to the Cochrane Handbook for Systematic Reviews of Interventions and the Preferred Reporting Items for Systematic Reviews and Meta-Analyses (or PRISMA) Statement guidelines^[19,20]^.

### Eligibility Criteria

Only fully published manuscripts meeting all the following eligibility criteria were included: (1) randomized controlled trials (RCTs); (2) including low-to-moderate surgical risk patients with severe AS; (3) comparing transcatheter *vs.* surgical aortic valve; (4) studies with follow-up ≥ 4 years; (5) availability of studies in English; and (6) reporting any of the clinical outcomes of interest. A minimum of a four-year follow-up was chosen based on the preliminary review of the literature which found substantial heterogeneity in follow-up between different studies (a few weeks to years). We excluded: (1) overlapping populations, defined as studies with overlapping institutions and recruitment periods; (2) non-randomized studies; (3) studies with no outcomes of interest; (4) conference abstracts; and (5) no control group. There were no restrictions based on the year of publication. In case of missing data from individual studies, the corresponding authors were contacted for specific study results.

### Search Strategy and Data Extraction

We systematically searched PubMed®, Embase, and Cochrane Central Register of Controlled Trials for RCTs meeting the eligibility criteria from inception to May 2024. The search strategy consisted of “(aortic valve replacement OR Aortic stenosis) AND (TAVI OR TAVR OR Aortic Transcatheter OR Transcatheter Aortic valve implantation) AND (Surgical Aortic valve replacement OR Surgical Aortic Valve Replacement OR Surgical Aortic Valve implantation)”. The references from all included studies, previous systematic reviews, and meta-analyses were also searched manually for any additional study^[21]^.

The search strategy was conducted by two authors (C.A.D.P. and C.A.P.P.). The studies found in the databases and the references of the articles were incorporated into the Rayyan reference management (Rayyan Systems Inc., Montreal, Canada). Duplicate articles were manually excluded. Any disagreements were resolved through consensus by the senior author (V.M.). The baseline characteristics were extracted by other two authors (H.S.F. and C.A.P.P). The outcome data following predefined search criteria and quality assessment was extracted by other two authors (C.A.D.P and L.F.T.).

### Endpoints and Subgroup Analyses

The main outcomes of interest were: (1) all-cause mortality and (2) disabling stroke. Subgroup analyses based on the participants’ surgical risk and the studies’ follow-up time were used to reduce heterogeneity.

### Quality Assessment

Quality assessment of RCTs was performed by two independent authors (A.J.F. and H.S.F.) using the Cochrane Collaboration’s tool for assessing risk of bias in RCTs (RoB 2), in which studies are scored as high, low, or unclear risk of bias in five domains: selection, performance, detection, attrition, and reporting biases^[22]^. Disagreements were resolved by the senior author (V.M.).

### Statistical Analysis

In order to compare treatment effects for categorical endpoints, a risk ratio (RR) with 95% confidence intervals (CI) was pooled using the Mantel-Haenszel method with the Der Simonian and Laird random-effects model. We assessed heterogeneity with I² statistics and Cochrane Q test; I² > 25% was considered significant for heterogeneity. *P*-values < 0.05 were considered statistically significant. Review Manager 5.1.7 (Cochrane Center, The Cochrane Collaboration, Denmark) and R software (version 4.3.2, R Foundation for Statistical Computing, Vienna, Austria) were used for statistical analysis. Aiming to explore the robustness of the results and identify outliers, leave-one-out sensitivity analyses were conducted by systematically removing each study from the research and recalculating the results for outcomes with significant heterogeneity.

## RESULTS

### Study Selection and Baseline Characteristics

**Fig. 1:**
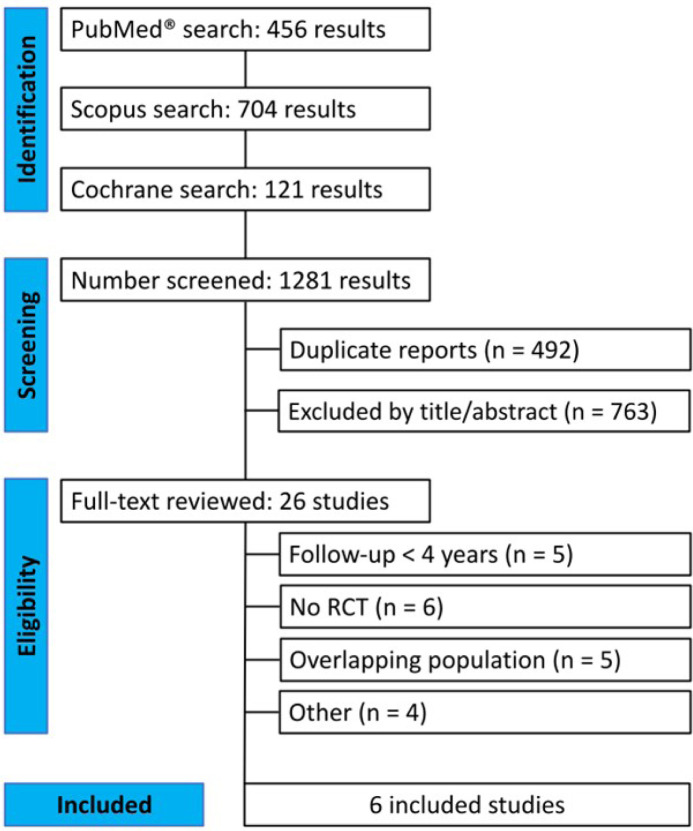
Preferred Reporting Items for Systematic Reviews and Meta-Analysis (or PRISMA) flow diagram of study screening selection. RCT=randomized controlled trial.

As detailed in Figure 1, the initial search yielded 1,281 results. After the removal of duplicate records and ineligible studies, 26 articles remained and were fully reviewed based on inclusion criteria. Of these, a total of six RCTs were included, comprising 6,498 patients^[23-28]^. Study characteristics are reported in Table 1. A total of 3,286 (51%) patients were treated with TAVR. The follow-up ranged between four and five years. The mean patient age was 78.4 years. There were 2,170 (66%) and 1,905 (59.3%) male patients in the TAVR and SAVR group, respectively. The mean Society of Thoracic Surgeons (STS) score of the included studies was 3.86.

**Table 1:** Baseline characteristics of included studies.

Study	Patients, n	Male, %	Age*, years	Follow-up, years	Country	NYHA III or IV, %	STS score*	DM, %
	TAVR/SAVR	TAVR/SAVR	TAVR/SAVR	TAVR/SAVR		TAVR/SAVR	TAVR/SAVR	TAVR/SAVR
SURTAVI 2022 ^[25]^	864/796	57.6/55	79.9 ± 6.2/79.7 ± 6.1	5	Europe and North America	60.1/58.1	4.4 ± 1.5/4.5 ± 1.6	34.3/34.8**
PARTNER 2020 ^[23]^	1011/1021	54.2/54.8	81.5 ± 6.7/81.7 ± 6.7	5	United States of America and Canada	77.3/76.1	5.8 ± 2.1/5.8 ± 1.9	37.7/34.2
NOTION 2019 ^[26]^	145/135	53.8/52.6	79.2 ± 4.9/79.0 ± 4.7	5	Denmark and Sweden	48.6/45.5	2.9 ± 1.6/3.1 ± 1.7	17.9/20.7
PARTNER 2023 ^[24]^	503/497	67.5/71.1	73.3 ± 5.83/73.6 ± 6.08	5	United States of America, Japan, Australia, and Canada	31.2/23.8	1.9 ± 0.7/1.9 ± 0.6	31.3/30.2
EVOLUT 2023 ^[27]^	734/734	915/64.7	74.0 ± 5.9/73.8 ± 6.0	4	Australia, Canada, France, Japan, The Netherlands, New Zealand, and United States of America	31.2/23.8	1.9 ± 0.7/1.9 ± 0.7	31.1/30.5
Rex 2016 ^[40]^	29/29	28/31	80 ± 4/82 ± 5	5	Denmark	24.6/27.9	3.2 ± 0.3/3.5 ± 0.2	0/10

Study	Previous pacemaker, %	Previous MI, %	Previous cerebrovascular events, %	Previous PCI, %	Previous CAD, %	Previous AF, %	PVD, %	CLD, %	Creatinine > 2 mg/dL, %	Transcatheter aortic valve model
	TAVR/SAVR	TAVR/SAVR	TAVR/SAVR	TAVR/SAVR	TAVR/SAVR	TAVR/SAVR	TAVR/SAVR	TAVR/SAVR	TAVR/SAVR	
SURTAVI 2022 ^[25]^	9.7/9.0	14.5/13.9	17.5/16.3	21.3/21.2	62.6/64.2	28.1/26.5	30.8/29.9	35.4/33.5	1.6/2.1	Medtronic supra-annular self-expanding bioprosthesis
PARTNER 2020 ^[23]^	11.7/12.0	18.3/17.5	32.1/31.0	27.1/27.6	69.2/66.5	31/35.2	27.9/32.9	31.8/30.	NA	Edwards SAPIEN 3 balloon-expandable heart valve system
NOTION 2019 ^[26]^	3.4/4.4	5.5/4.4	16.6/16.3	7.6/8.9	NA	27.8/25.6^‡^	4.1/6.7	11.7/11.9	1.4/0.7	Medtronic Mosaic^™^, Carpentier-Edwards PERIMOUNT, Sorin Mitroflow, and first-generation self-expanding Medtronic CoreValve^™^
PARTNER 2023 ^[24]^	2.4/2.9	5.7/5.8	NA	NA	27.7/28	15.7/18.8	6.9/7.3	5.1/6.2	0.2/0.2	Edwards SAPIEN 3 balloon-expandable heart valve system
EVOLUT 2023 ^[27]^	3.4/3.8^$^	6.7/5.3	10.1/11.4	13.9/12.7	NA	15.5/14.9	7.6/8.5	15.1/17.2	0.4/0.1	Medtronic supra-annular self-expanding bioprosthesis
Rex 2016 ^[40]^	NA	NA	NA	NA	NA	NA	NA	3-out.	NA	Edwards SAPIEN 3 balloon-expandable heart valve system

* Data are presented as mean ± standard deviation;

** The article did not specify the type of diabetes

DM=diabetes mellitus; NYHA=New York Heart Association; SAVR=surgical aortic valve replacement; STS=Society of Thoracic Surgeons; TAVR=transcatheter aortic valve replacement

‡ The article included atrial fibrillation and atrial flutter in the same data;

$ The article included pacemaker and defibrillator in the same data

AF=atrial fibrillation; CAD=coronary artery disease; CLD=chronic lung disease; MI=myocardial infarction; NA=data not available; PCI=percutaneous coronary intervention; PVD=peripheral vascular disease; SAVR=surgical aortic valve replacement; TAVR=transcatheter aortic-valve replacement

### Pooled Analyses of All Studies

**Fig. 2:**
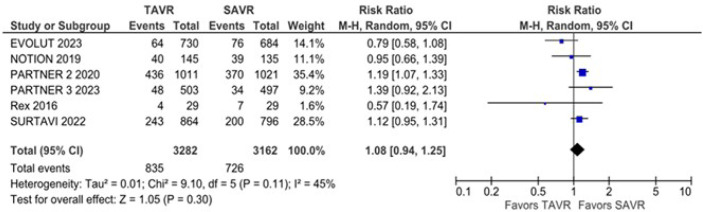
All-cause mortality was not significantly different between surgical aortic valve replacement (SAVR) and transcatheter aortic valve replacement (TAVR). CI=confidence interval.

**Fig. 3:**
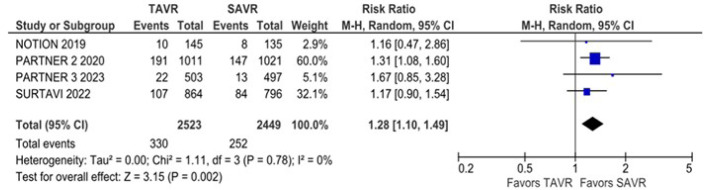
Disabling stroke was not significantly different between groups. CI=confidence interval; SAVR=surgical aortic valve replacement; TAVR=transcatheter aortic valve replacement.

**Supplementary Figure 1:**
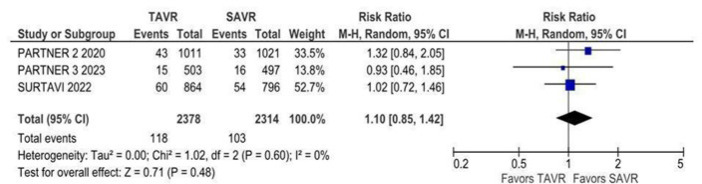
Forest plot for endocarditis comparing transcatheter aortic valve replacement (TAVR) with surgical aortic valve replacement (SAVR). CI=confidence interval.

There was no statistically significant difference between groups in all-cause mortality (RR 1.08; 95% CI 0.94 - 1.25; *P* = 0.30; I^2^ = 45%) (Figure 2), cardiovascular mortality (RR 1.09; 95% CI 0.96 - 1.23; *P* = 0.17; I^2^ = 0%), stroke (RR 1.04; 95% CI 0.85 - 1.26; *P* = 0.73; I^2^ = 18%), disabling stroke (RR 0.95; 95% CI 0.75 - 1.21; *P* = 0.67; I^2^ = 9%) (Figure 3), non-disabling stroke (RR 1.10; 95% CI 0.85 - 1.42; *P* = 0.71; I^2^ = 0%), endocarditis (RR 1.33; 95% CI 0.85 - 2.09; *P* = 0.21; I^2^ = 0%) (Supplementary Figure 1), myocardial infarction (RR 1.11; 95% CI 0.76 - 1.63; *P* = 0.58; I^2^ = 50%), and rehospitalization (RR 1.07; 95% CI 0.85 - 1.36; *P* = 0.55; I^2^ = 76%).

**Fig. 4:**
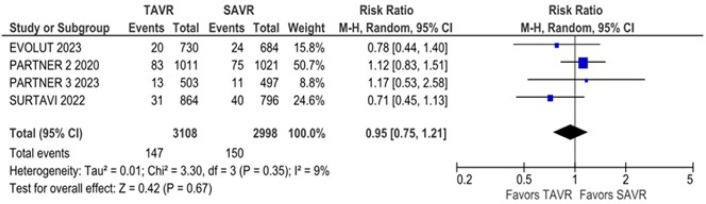
Non-cardiovascular mortality was significantly higher in the transcatheter aortic valve replacement (TAVR) group. CI=confidence interval; SAVR=surgical aortic valve replacement.

**Fig. 5:**
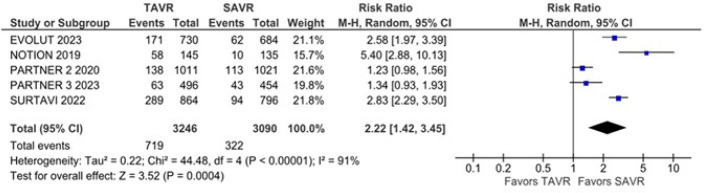
New pacemaker implantation was significantly higher in the transcatheter aortic valve replacement (TAVR) group. CI=confidence interval; SAVR=surgical aortic valve replacement.

**Fig. 6:**
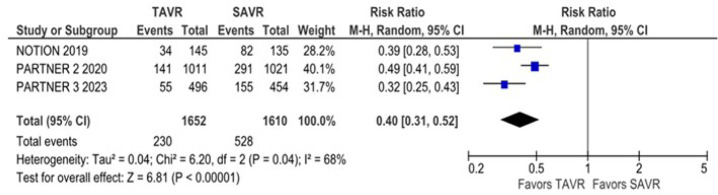
New atrial fibrillation was significantly lower in the transcatheter aortic valve replacement (TAVR) group. CI=confidence interval; SAVR=surgical aortic valve replacement.

Non-cardiovascular mortality was significantly higher in the TAVR group compared with the SAVR group (RR 1.28; 95% CI 1.10 - 1.49; *P* = 0.002; I^2^ = 0%) (Figure 4). The rate of new pacemaker implantation was also significantly higher in the TAVR group compared with the SAVR group (RR 2.22; 95% CI 1.42 - 3.45; *P* = 0.0004; I^2^ = 91%) (Figure 5). New atrial fibrillation was significantly lower in the TAVR group compared with the SAVR group (RR 0.40; 95% CI 0.31 - 0.52; *P* = 0.00001; I^2^ = 68%) (Figure 6).

### Subgroup Analysis

**Fig. 7:**
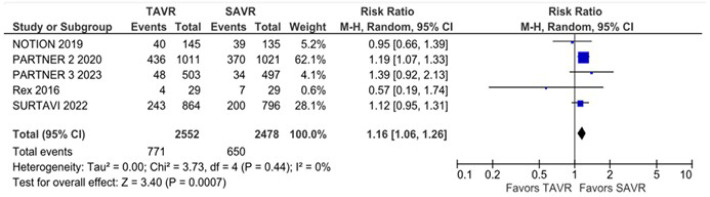
Subanalysis of studies with five years of follow up, all-cause mortality was significantly higher in the transcatheter aortic valve replacement (TAVR) group. CI=confidence interval; SAVR=surgical aortic valve replacement.

The risk of all-cause mortality was significantly higher in the TAVR group compared with the SAVR group over five years of follow-up (RR 1.28; 95% CI 1.10 - 1.49; *P* = 0.002; I^2^ = 0%) (Figure 7). There was no statistically significant difference in all-cause mortality between TAVR and SAVR in patients at low surgical risk (RR 0.96; 95% CI 0.71 - 1.29; *P* = 0.77; I^2^ = 44%).

**Supplementary Figure 2:**
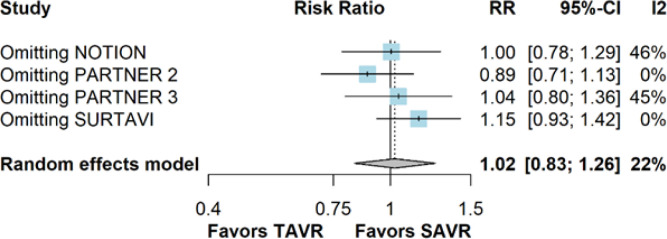
Leave-one-out analysis for stroke. CI=confidence interval; RR=risk ratio; SAVR=surgical aortic valve replacement; TAVR=transcatheter aortic valve replacement.

**Supplementary Figure 3:**
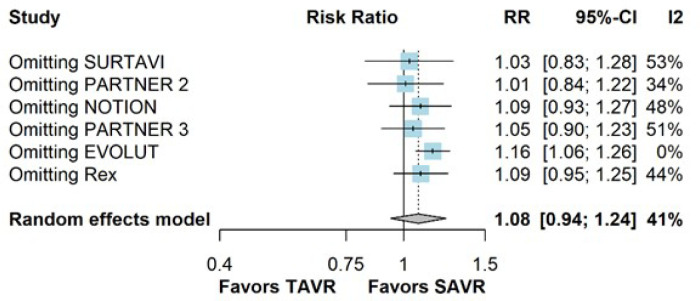
Leave-one-out analysis for all-cause mortality. CI=confidence interval; RR=risk ratio; SAVR=surgical aortic valve replacement; TAVR=transcatheter aortic valve replacement.

The leave-one-out analysis demonstrated the robustness of the pooled results for stroke (Supplementary Figure 2). However, the leave-one-out analysis for all-cause mortality showed that the omission of the EVOLUT trial reduced heterogeneity to 0% and led to statistically significant results (Supplementary Figure 3).

### Quality Assessment

Individual RCT appraisal is reported in Supplementary Table 1. We used version 2 of the Cochrane Risk of Bias assessment tool (RoB 2) to assess the individual overall risk of bias publication of the RCTs in this meta-analysis. Two studies were classified as low risk of bias, whereas three studies were evaluated as having some concerns in risk of bias mainly due to deviations from intended interventions. One study was evaluated at high risk due to the selection of the reported results, as the article did not report all outcomes pre-specified in its protocol (Supplementary Table 1).

High rates of heterogeneity were present in this analysis for outcomes, such as all-cause mortality, myocardial infarction, and rehospitalization. The present variation in data is possible due to the different types of prostheses used in each study, variability in healthcare settings, variability in medical expertise (years of practice), differences in patients-associated comorbidities, and inclusion of studies with less methodological rigor marked as “some concerns” or “high concerns” for risk of bias.

## DISCUSSION

This systematic review with meta-analysis of RCTs comprising > 6,000 low-to-moderate risk patients with severe AS compared mid-term outcomes between SAVR and TAVR. Our main findings were: (1) there was no significant difference between groups in terms of all-cause mortality, stroke, endocarditis, myocardial infarction, and rehospitalization; (2) there was a higher risk of non-cardiovascular death and new pacemaker implantation in the TAVR group compared with the SAVR group; and (3) there was a reduced risk of atrial fibrillation in the TAVR group when compared with the SAVR group.

Despite the established efficacy and safety of TAVR in high-risk cases, the extension of its application to those with lower or intermediate surgical risk requires a thorough evaluation of outcomes in mid and long-term follow-up. Our results suggest that the use of TAVR over a mid-term follow-up showed similar risk in all-cause mortality and stroke rates when compared with SAVR in patients with AS. These findings align with results from previous meta-analyses with shorter follow-up^[17,18,29–31]^. In the PARTNER 2 trial, there was no significant difference in all-cause mortality or disabling stroke when compared with SAVR^[23]^. These findings were subsequently corroborated by the PARTNER 3, SURTAVI, and NOTION trials^[24-26]^, all of which confirmed no significant differences between the groups for all-cause mortality.

A recent meta-analysis including low-surgical-risk patients demonstrated a reduction in the risk of all-cause mortality and disabling stroke at one year in the TAVR group. However, a mid-term analysis with an average follow-up of 4.3 years showed no difference between the groups for these same outcomes^[32]^. Nevertheless, a subgroup analysis of studies with five years of follow-up showed a significantly higher risk of all-cause mortality in the TAVR group when compared with the SAVR group. These findings are consistent with the PARTNER 2 study, which showed an increased risk of all-cause mortality in the TAVR group when compared with the SAVR group^[23]^. These different results between the individual studies may be explained by the use of different transcatheter systems that present different clinical performance and durability. The device (SAPIEN XT) used in the PARTNER 2 study is no longer in clinical practice and it was related to higher mortality and neurological events in the medium and long term when compared with other newer devices^[23,33]^. Therefore, our results of all-cause mortality over five years may be justified by the durability of the prostheses used in the TAVR group.

In addition, our leave-one-out sensitivity analysis for all-cause mortality showed that omitting the EVOLUT trial significantly reduced heterogeneity from 40% to 0%, with a statistically significant difference unfavoring TAVR when compared with SAVR in patients with AS and low to moderate surgical risk. The EVOLUT trial demonstrated the greatest benefit of TAVR over SAVR for all-cause mortality among included studies^[27]^. This may be explained by the use of a high-performance valve with advanced technology and higher loss to follow-up in the SAVR group. A previous meta-analysis compared mid-term outcomes between the two techniques including low, intermediate, and high-risk patients, and it showed that the advantages of TAVR over SAVR are not consistent over time, with longer follow-up revealing results favoring surgery^[34]^.

Increased survival rates with TAVR in high-risk patients is largely due to reduced cardiovascular mortality^[16]^. However, non-cardiovascular and non-categorizable causes contributed significantly to the mortality of these patients. Our findings suggest a higher risk of death from non-cardiovascular causes for TAVR when compared with SAVR in patients with AS and low to moderate surgical risk (RR 1.28). Although several clinical trials showed a greater number of deaths from non-cardiovascular causes in the TAVR group when compared with the SAVR group in patients with AS and low to moderate surgical risk, these results were not statistically significant^[25,26]^. A previous meta-analysis identified infections/sepsis as the leading cause of non-cardiovascular death within 30 days and the second cause of death after 30 days^[30]^. Although TAVR is a minimally invasive procedure, patients generally present factors alone or in combination that predispose to infection, including age; poor lung, kidney, and immune function; diabetes; and need for ventilation and central venous access and monitoring^[35]^.

Our analysis revealed no difference in rehospitalization between the TAVR and SAVR groups in patients with AS and low to moderate surgical risk. In the PARTNER 3 study, 1,000 patients with severe AS and low surgical risk were randomized to TAVR or SAVR. In the intention-to-treat analysis during five years of follow-up, there was no difference in rehospitalization between the groups^[24]^. The same results were found in the SURTAVI study, which showed no difference in readmission between the groups^[26]^. However, these results differ from the PARTNER 2 study, which showed a higher risk of readmission for TAVR when compared to SAVR at five years in patients with AS and low to moderate surgical risk^[23]^. Previous literature identified valve stenosis or regurgitation for TAVR and endocarditis for SAVR as the main causes of hospitalization^[36]^. Our findings may be explained by the fact that the studies used different devices for TAVR. Different transcatheter models are associated with different risks of complications, often requiring reintervention^[26]^.

The findings of this systematic review with meta-analysis show a higher risk of pacemaker implantation in the TAVR group when compared with SAVR in patients with AS and low to moderate surgical risk, which is consistent with previously published literature^[17,26,27,37]^. Despite the high risk of pacemaker implantation in the TAVR group, this risk varies between different studies, from 5.40 to 1.23. The NOTION study was the first RCT to study low-risk patients and it was the study with the highest risk of pacemaker implantation^[26]^. Subsequent studies showed an increasingly lower risk of pacemaker implantation^[23,25,27]^. The PARTNER 2 and 3 trials showed the lowest risks for pacemaker implantation^[23,24]^. Several factors may influence this variation in risks, such as studies using different devices for TAVR. In the PARTNER studies, different from the NOTION study, third-generation devices were used. SAPIEN 3, which is the latest transcatheter heart valve in the Edwards family, incorporates a number of new and improved features and it also appeared to have a more favorable clinical profile in terms of clinical and valve performance with fewer complications, including a lower risk of implanting a new pacemaker^[38]^.

Literature has shown differences in clinical outcomes when comparing different transcatheter valve systems with surgical valves. The second-generation balloon-expandable transcatheter valve has a higher risk of structural valve degeneration than the surgical valve^[38,39]^. The third-generation balloon-expandable transcatheter heart valve (SAPIEN 3, Edwards Lifesciences) appeared to have a more favorable clinical profile in terms of clinical outcomes and valve performance^[33]^.

Our results showed a reduced incidence of atrial fibrillation in the TAVR group when compared with the SAVR group in patients with AS and low to moderate surgical risk. In the PARTNER 2 study, 2023 patients with symptomatic severe AS and intermediate surgical risk were randomized to TAVR or SAVR and followed for five years. The risk of atrial fibrillation was twice as high in the SAVR group when compared with the TAVR group, and the same results were reported in previous literature. Other statistically non-significant results from our analyses also deserve comment: TAVR may not be associated with fewer outcomes of endocarditis and myocardial infarction when compared with SAVR in a medium follow-up period, and these results are consistent with previous reports^[23,24,26]^.

SAVR is a safe technique with significantly low operative mortality in selected elderly patients, but it increases with the number and severity of comorbidities, imposing an important limitation on SAVR^[10]^. Medium- and long-term outcomes vary widely, with survival rates ranging from 37.4% to 64%^[40-42]^. These results are significantly influenced by the patient’s age and the presence of comorbidities. Structural valve degeneration, which limits its durability, represents the main limitation of biological tissue^[10]^. It is evident that following the advent of TAVR, SAVR outcomes have significantly improved, likely because higher-risk patients were increasingly referred for TAVR^[43]^. TAVR is a minimally invasive and safe technique, but certain complex anatomical characteristics such as the access site, pathway, and valve implantation site can hinder its successful use or even contraindicate TAVR^[44]^. Although initially tested in high-surgical-risk patients, its use has expanded to those with moderate and even low risk. Due to its minimally invasive nature, TAVR avoids sternotomy and cardiopulmonary bypass, potentially reducing resource utilization by accelerating patient recovery and shortening hospital stays^[45]^. The NOTION study recruited participants in the early 2010s and used the self-expanding CoreValve™ system, showing a similar risk of the composite endpoint for TAVR and SAVR at five and eight years. Among studies with mid-term follow-up, the EVOLUT Low Risk trial demonstrated a lower risk of all-cause mortality in the TAVR group (6.3%) when compared to the SAVR group (12.4%). This study utilized self-expanding aortic valves, Evolut™ R and Evolut™ PRO. In the PARTNER 3 trial, which investigated the balloon-expandable SAPIEN 3 valve, the four-year mortality rate was slightly higher in the TAVR group (7.4%) when compared to the SAVR group (5.9%)^[24,27,38]^.

The overall composite endpoint rate in the NOTION study was higher than in the EVOLUT LOW RISK and PARTNER 3 studies. This is likely related to a higher mean STS score among NOTION participants, as well as the use of non-contemporary valve technology and medical therapy. Recently, a systematic review and network meta-analysis compared different transcatheter heart valves with SAVR. The study showed a similar risk of all-cause mortality among the groups. However, the risk of disabling stroke was lower with mechanically expandable valves when compared to balloon-expandable valves and SAVR, and it was also lower with self-expanding valves when compared to SAVR in the long term. On the other hand, mechanically expandable valves were associated with a higher risk of pacemaker implantation when compared to other systems and SAVR^[46]^.

### Limitations

This systematic review with meta-analysis has some limitations. Most importantly, none of the studies were blinded — a fundamental limitation arising from the nature of the interventions. There was, also, some variability in the follow-up time between studies. To minimize such heterogeneities, we performed subgroup analysis in studies comparing TAVR *vs.* SAVR with a follow-up higher than four years. Furthermore, there were important differences in the types of prostheses used in the studies. This difference, unfortunately, can impact negatively on the clinical applicability of our results across diverse contexts. Finally, significant heterogeneity was found in the outcome of all-cause mortality. However, the leave-one-out sensitivity analysis showed the robustness of the overall findings.

## CONCLUSION

The results of this meta-analysis, including over 6,000 patients with AS and low to moderate surgical risk, suggest TAVR is non-inferior to SAVR regarding all-cause mortality or stroke in the mid-term period. Although both procedures are safe, the choice of treatment must be individualized and made together with the patient and the heart valve team.

## Data Availability

The authors declare that data sharing is not applicable to this article as it is a meta-analysis and no new data were created or analyzed.

## Appendix

**Supplementary Table 1:** Critical appraisal of individual studies according to the Cochrane Collaboration’s tool for assessing risk of bias in randomized trials.

Study	Bias from randomization process	Bias due to deviations from intended interventions	Bias due to missing outcome data	Bias in measurement of the outcomes	Bias in selection of the reported result	Overall risk of bias
SURTAVI 2022 ^[25]^	Low	Low	Low	Low	High	High
PARTNER II 2020 ^[23]^	Low	Low	Low	Low	Low	Low
NOTION 2019 ^[26]^	Low	Some concerns	Low	Low	Low	Some concerns
PARTNER III 2023 ^[24]^	Low	Some concerns	Low	Low	Low	Some concerns
EVOLUT 2023 ^[27]^	Low	Low	Some concerns	Low	Low	Some concerns
Rex 2016 ^[40]^	Low	Low	Low	Low	Low	Low
